# IFN-γ–STAT1–iNOS Induces Myeloid Progenitors to Acquire Immunosuppressive Activity

**DOI:** 10.3389/fimmu.2017.01192

**Published:** 2017-09-22

**Authors:** Shu-Han Yang, Liang Li, Yu-Qing Xie, Yuan Yao, Cai-Yue Gao, Liang-Huan Liao, Hong-Di Ma, M. Eric Gershwin, Zhe-Xiong Lian

**Affiliations:** ^1^Liver Immunology Laboratory, School of Life Sciences, Institute of Immunology, University of Science and Technology of China, Hefei, China; ^2^Chronic Disease Laboratory, School of Medicine, Institutes for Life Sciences, South China University of Technology, Guangzhou, China; ^3^Division of Rheumatology, Allergy and Clinical Immunology, University of California at Davis School of Medicine, Davis, CA, United States; ^4^Innovation Center for Cell Signaling Network, Hefei National Laboratory for Physical Sciences at Microscale, Hefei, China

**Keywords:** immunosuppression, autoimmune disease, myeloid progenitors, T cells, bone marrow, IFN-γ, STAT1, inducible nitride oxide synthase

## Abstract

Autoimmune diseases often induce dysregulated hematopoiesis with altered number and function of hematopoietic stem and progenitor cells (HSPCs). However, there are limited studies on the direct regulation of HSPCs on T cells, which are often detrimental to autoimmunity. Here, we found that in a murine model of Concanavalin A-induced autoimmune hepatitis, LSK (Lineage^−^Sca-1^+^c-Kit^+^)-like cells accumulated in liver, spleen, and bone marrow (BM), which were myeloid progenitors (Lineage^−^Sca-1^−^c-Kit^+^) that upregulated Sca-1 expression upon T cell-derived IFN-γ stimulation. Strikingly, BM LSK-like cells from mice induced by Con A to develop autoimmune hepatitis or alternatively myeloid progenitors from wild-type mice possessed strong *in vitro* suppressive ability. Their suppressive function depended on T cell-derived IFN-γ in a paracrine fashion, which induced STAT1 phosphorylation, inducible nitric oxide synthase expression, and nitric oxide production. Blocking IFN-γ/IFN-γ receptor interaction, knockout of STAT1, or iNOS inhibition abrogated their suppressive function. In addition, the suppressive function was independent of differentiation; mitomycin C-treated myeloid progenitors maintained T cell suppressive ability *in vitro*. Our data demonstrate a mechanism of inflammation induced suppressive function of myeloid progenitors, which may participate directly in suppressing T cell-mediated immunopathology.

## Introduction

Hematopoietic stem and progenitor cells (HSPCs) are a population of stem cells in the bone marrow (BM), which can give rise to all lineages of blood cells including leukocytes. These cells are identified by surface markers as Lineage^−^Sca-1^+^c-Kit^+^, coined LSK cells, and undergo differentiation following a hierarchy from long-term-HSCs (LT-HSCs) to short-term-HSC (ST-HSCs) to multipotent progenitors (MPPs) (1, 2). LT-HSCs have the capacity to self-renew over the lifespan, while ST-HSCs and MPPs have less self-renewal capacity and can differentiate into committed progenitors like common lymphoid progenitors and common myeloid progenitors (CMPs), which replenish hematopoietic populations including lymphoid cells and myeloid cells when they are depleted or reduced by aging or consumed (3, 4).

Hematopoietic stem and progenitor cells usually reside in a quiescent state within a niche of microenvironment in BM, which generate signals that regulate HSC self-renewal, quiescence, and differentiation (5). During infection and inflammation, HSPCs can be activated, mobilized, and differentiate to help against pathogens, as well as inflammation resolution (6, 7). HSC transplantation has been used for decades in autoimmune disease treatment to replace autoreactive cells (8). HSPCs traffic to liver and differentiate into alternatively activated macrophages in a CCR2-dependent way to protect against acetaminophen-induced liver injury (9). Blood-derived CD34^+^ progenitor cells can rescue mice from severe acute liver injury and induce liver regeneration (10). However, in a murine model of IL-23-dependent colitis, intestinal inflammation increases hematopoietic stem and progenitor cell proliferation and skews their differentiation toward granulocyte-monocyte progenitors (GMP), leading to aggravation of colitis (11). Thus, HSPCs play a controversial role during acute or chronic inflammation, whether they can interact directly with T cells to modulate inflammation remains unclear.

Autoimmune diseases often induce a dysregulated hematopoiesis (12, 13). Hematopoietic progenitor cells from patients with autoimmune hepatitis are significantly increased in number but functionally impaired (14). Hence, we chose the model of Con A-induced AIH to dissect the direct interaction between HSPCs and T cells. We report that LSK-like cells accumulate in BM, liver, and spleen after Con A treatment; LSK-like cells are myeloid progenitor cells, which upregulate Sca-1 expression during activation. Most importantly, these HSPCs possess powerful T cell suppressive ability *in vitro* and their suppressive function is acquired following T cell-derived IFN-γ stimulation, which induces STAT-1 phosphorylation, iNOS expression, and NO production, but is independent of differentiation. Our data demonstrate that myeloid progenitor cells acquire T cell suppressive activity using an IFN-γ–STAT1–iNOS pathway.

## Materials and Methods

### Mice

C57BL/6 mice were purchased from Shanghai Laboratory Animal Center, Chinese Academy of Science (Shanghai, China). IFN-γ^−/−^ (B6.129S7-IFN-γ^tm1Ts^/J), IFN-γR1^−/−^ (B6.129S7-Ifngr1^tm1Agt^/J), OT-II transgenic (B6.Cg-Tg (TcraTcrb) 425Cbn/J), H2Ab1^−/−^ (B6.129S2-*H2*^dlAb1-Ea^/J), Rag1^−/−^ and CD45.1 congenic mice on a C57BL/6 background were initially obtained from the Jackson Laboratory (Bar Harbor, ME, USA). OT-II mice were crossed with CD45.1 mice or IFN-γ^−/−^ mice to generate CD45.1 OT-II or IFN-γ^−/−^ OT-II (GKO OT-II) mice. STAT1^−/−^ mice on a C57BL/6 background were kindly provided by Dr. Rongbin Zhou from University of Science and Technology of China (USTC). All mice were housed under specific pathogen-free condition at the Laboratory Animal Center, USTC, and handled in accordance with the institution’s guidelines. Animal experiments were performed following approval of the USTC Animal Care and Use Committee.

### Autoimmune Hepatitis Model

10-week-old male mice were injected i.v. with Con A (10 mg/kg body weight, Sigma-Aldrich, St. Louis, MO, USA) and sacrificed 24 h later or at indicated time points. Serum alanine aminotransferase (ALT) level was quantitated to evaluate liver damage.

### Tissue Processing and Flow Cytometry

Single cell suspension from spleen and BM were prepared as previously described (15). Livers were homogenized and mononuclear cells were separated from hepatocytes by centrifugation with 40% Percoll (GE Healthcare, Little Chalfont, United Kingdom). A 1–1.5 × 10^6^ cells aliquot was blocked with anti-mouse CD16/32 (93) (BioLegend, San Diego, CA, USA) and then stained with a mixture of fluorochrome-conjugated monoclonal antibodies including CD69 (H1.2F3), CD19 (6D5), CD4 (GK1.5), CD8α (53-6.7), Sca-1 (D7), c-Kit (2B8), I-A/I-E (M5/114.15.2), CD80 (16-10A1), CD86 (GL-1), CD16/32 (93), CD45.1 (A20), CD45.2 (104), CD34 (RAM34), PD-L1(10F.9G2), and antibodies against lineage markers: CD11c (N418), CD3 (17A2), Ter119, Gr-1 (RB6-8C5), NK1.1 (PK136), B220 (RA3-6B2), CD11b (M1/70). Ki67 (16A8, Biolegend) was detected using Foxp3/Transcription Factor Staining Buffer Set (eBioscience, San Diego, CA, USA). Cells were subjected to FACSVerse flow cytometer (BD Immunocytometry Systems, San Jose, CA, USA) and data were analyzed with Flowjo software (Tree Star Inc., Ashland, OR, USA).

### Cell Enrichment and Sorting

Mononuclear cells from BM were prepared as described in sterile PBS containing 2.5% fetal bovine serum (FBS) (Millipore, Darmstadt, Germany) and 100 U/ml Penicillin/Streptomycin (Hyclone, Logan, UT, USA).

Lineage negative (Lin^−^) cells were enriched with a Lineage Cell Depletion Kit (Miltenyi Biotec Inc., Bergisch Gladbach, Germany) by magnetic-activated cell sorting (MACS) and stained with antibodies (BioLegend) as PE or PE/Cy7 conjugated [CD4 (GK1.5), CD8α (53-6.7), CD19 (6D5), NK1.1 (PK136), Gr-1 (RB6-8C5), Ter119], FITC-Sca-1 (D7), PE/Cy5-c-Kit (2B8), FITC-CD34 (RAM34), PE-CD16/32 (93), APC/Cy7-Sca-1 (D7). LSK (Lin^−^Sca-1^+^c-Kit^+^) cells, myeloid progenitors (Lin^−^Sca-1^−^c-Kit^+^), CMP (Lin^−^Sca-1^−^c-Kit^+^CD34^+^CD16/32^mid^), GMP (Lin^−^Sca-1^−^c-Kit^+^CD34^+^CD16/32^hi^), megakaryocyte–erythrocyte progenitor (MKEP) (Lin^−^Sca-1^−^c-Kit^+^CD34^−^CD16/32^lo^) sorting was performed with a MoFlo^®^ Astrios^EQ^ (Beckman Coulter, Brea, CA, USA).

OT-II or GKO OT-II CD4^+^ T cells from spleens were positively selected by MACS using Mouse CD4 (L3T4) MicroBeads (Miltenyi Biotec Inc.). Wild type (WT) or IFN-γ^−/−^ (GKO) T cells (CD3^+^CD25^−^), B cells (CD3^-^CD19^+^), monocytic myeloid cells (CD11b^+^Ly-6G^−^Ly-6C^+/hi^), and granulocytic myeloid cells (CD11b^+^Ly-6G^+/hi^) from spleen or BM were sorted by MoFlo^®^ Astrios^EQ^ (Beckman Coulter). Sorting purities using MACS and FACS were greater than 90 and 95%, respectively.

### *In Vitro* HSPC Culture

5 × 10^4^/well WT or IFN-γR^−/−^ (GRKO) myeloid progenitor cells were cocultured with 5 × 10^4^/well WT or GKO OT-II T cells in the presence of Con A (2 µg/ml) for 24 h. For IFN-γ stimulation assay, 5 × 10^4^/well WT, GRKO, or STAT1^−/−^ myeloid progenitor cells were cultured in the presence of 20 ng/ml IFN-γ for 24 h.

Cells were cultured in T cell medium [RPMI 1640 (Gibco, Waltham, MA, USA) supplemented with 10% FBS (Millipore), 2 mM l-glutamine (Gibco), 1 mM sodium pyruvate (Gibco), 25 mM HEPES-free acid (Gibco), 55 µM 2-mercaptoethanol (Gibco), and 100 U/ml Penicillin/Streptomycin (Hyclone)] in a 96-well round bottom plate (Corning, NY, USA).

After culture, dead cells were excluded by DAPI staining and phenotype of HSPCs was analyzed by flow cytometry.

### *In Vitro* T Cell Suppression

For antigen-specific suppression assays, 1 × 10^4^/well HSPCs from mice treated with Con A for 24 h or WT myeloid cells were cocultured with 5 × 10^4^/well carboxyfluorescein succinimidyl ester (CFSE) (2 µM, Life Technologies, Waltham, MA, USA) labeled OT-II or GKO OT-II T cells for 72 h, in the presence of 1 µg/ml Ovalbumin peptide 323–339 (OVA_323–339_) (Sigma-Aldrich), and 1 × 10^4^/well B cells as supporters. To evaluate the suppressive ability of HSPCs, the number of WT myeloid progenitors or Con A LSK cells was reduced at different gradient as HSPC:T = 1:5/10/20/50. HSPCs and LSK cells were from BM unless indicated.

PD-L1 blockade antibody (10F.9G2, Biolegend, 5 µg/ml) was used to block PD-L1-PD-1 signaling (16), and LEAF™ Purified IgG2b, κ (Biolegend) antibody was used as isotype control.

In some experiments, HSPCs were treated with 25 µg/ml Mitomycin C (Sigma) for 30 min at 37°C and washed for at least five times before adding to the coculture system; Mitomycin C-treated B cells were used as control.

For mixed proliferation experiment, 5 × 10^4^/well non-CFSE-labeled OT-II T cells were added into the coculture system of WT myeloid progenitors and GKO OT-II T cells, while 5 × 10^4^/well non-CFSE-labeled GKO OT-II T cells were added as control. Proliferation of CFSE^+/lo^ GKO OT-II T cells was evaluated.

Cells were cultured in T cell medium. After culture, dead cells were excluded by DAPI staining and T cell proliferation was assessed by CFSE dilution of B220^−^CD4^+^ cells. Percentage of proliferation was normalized by the control system.

### *In Vitro* HSPC Proliferation and Differentiation Assay

5 × 10^4^/well CFSE-labeled WT myeloid progenitor cells were cocultured with 5 × 10^4^/well non-CFSE-labeled WT or GKO OT-II T cells in the presence of 1 µg/ml OVA_323–339_ for 24/48/72 h. Proliferation and differentiation of HSPCs was evaluated by CFSE dilution and CD11b/Gr-1 expression of DAPI^−^B220^−^CD4^−^ cells.

### *In Vitro* Nitric Oxide Inhibition

Representative nitric oxide synthase (NOS) inhibitors (Beyotime, Jiangsu, China) including l-NMMA (Pan NOS inhibitor, 200 µM), 1,400 W (iNOS inhibitor, 100 µM), and L-NAME (eNOS inhibitor, 100 µM) were used in *in vitro* T cell suppression experiments to inhibit the generation of NO.

### Transwell Assay

For transwell assays, 2.5 × 10^5^ CFSE-labeled OT-II T cells and 5 × 10^4^ B cells with or without 5 × 10^4^ WT myeloid progenitors were cultured in the top or bottom chamber of Corning Transwell-96 System (0.4 µm PC membrane, corning, NY, USA) for 3 days in the presence of 1 µg/ml OVA_323–339_ peptide. Cells were collected respectively and proliferation of DAPI^−^CD4^+^T cells was analyzed by CFSE dilution.

### Cytometric Bead Array

Concentrations of IFN-γ in serum from acute hepatitis mice/control mice were measured with a cytometric bead array kit (Mouse Th1/Th2/Th17 CBA kit, BD Biosciences) and analyzed using a FACS Verse flow cytometer with CBA software (BD Biosciences).

### Giemsa Staining

1 × 10^4^ purified HSPCs were centrifuged on a cover glass in Cytocentrifuge Hettich Universal 32 (Hettich, Tuttlingen, Germany), followed by Wright-Giemsa Staining (SolarBio, Beijing, China).

### Western Blotting

3 × 10^5^ purified HSPCs were lysed in sample buffer [50 mM Tris–HCl, pH 7.4, 0.15 mM Bromophenol Blue, and 10% (vol/vol) glycerol]. Proteins were separated by sodium dodecylsulfate-polyacrylamide gel electrophoresis and transferred to Immobilon-P PVDF Membrane (Millipore). After blocking with 5% non-fat milk, membranes were stained with p-STAT1 (Tyr701) (58D6) Rabbit mAb (Cell Signaling Technology, MA, USA), GAPDH Mouse antibody (GeneSci, Beijing, China), and detected by HRP-labeled anti-rabbit (CST) and anti-mouse antibody (Beyotime), respectively.

### Real-time PCR

Total RNA was extracted with RNAiso Plus (TaKaRa Bio Inc., Shiga, Japan). Genome DNA was removed and reverse transcription was performed using PrimeScript RT Reagent Kit with gDNA Eraser (TaKaRa). Quantitative real-time PCR was performed using SYBR Premix Ex Taq II (TaKaRa). Primers included iNOS forward: CGA AACGCTTCACTTCCAA, reverse: TGAGCCTATATTGCTGTG GCT; GAPDH forward: CATGGCCTTCCGTGTTCCTA, reverse: CCTGCTTCACCACCTTCTTGAT.

### Statistical Analysis

For analysis of statistical significance, we performed a two-tailed unpaired Student’s *t*-test in GraphPad Prism 5 (GraphPad Software, San Diego, CA, USA). Data in all results were from at least two independent experiments and were shown as mean ± SEM.

## Results

### T Cell-Derived IFN-γ Induces LSK (Lin^−^Sca-1^+^c-kit^+^)-Like Cells from Myeloid Progenitors during Con A-Induced Autoimmune Hepatitis

**Figure 1:**
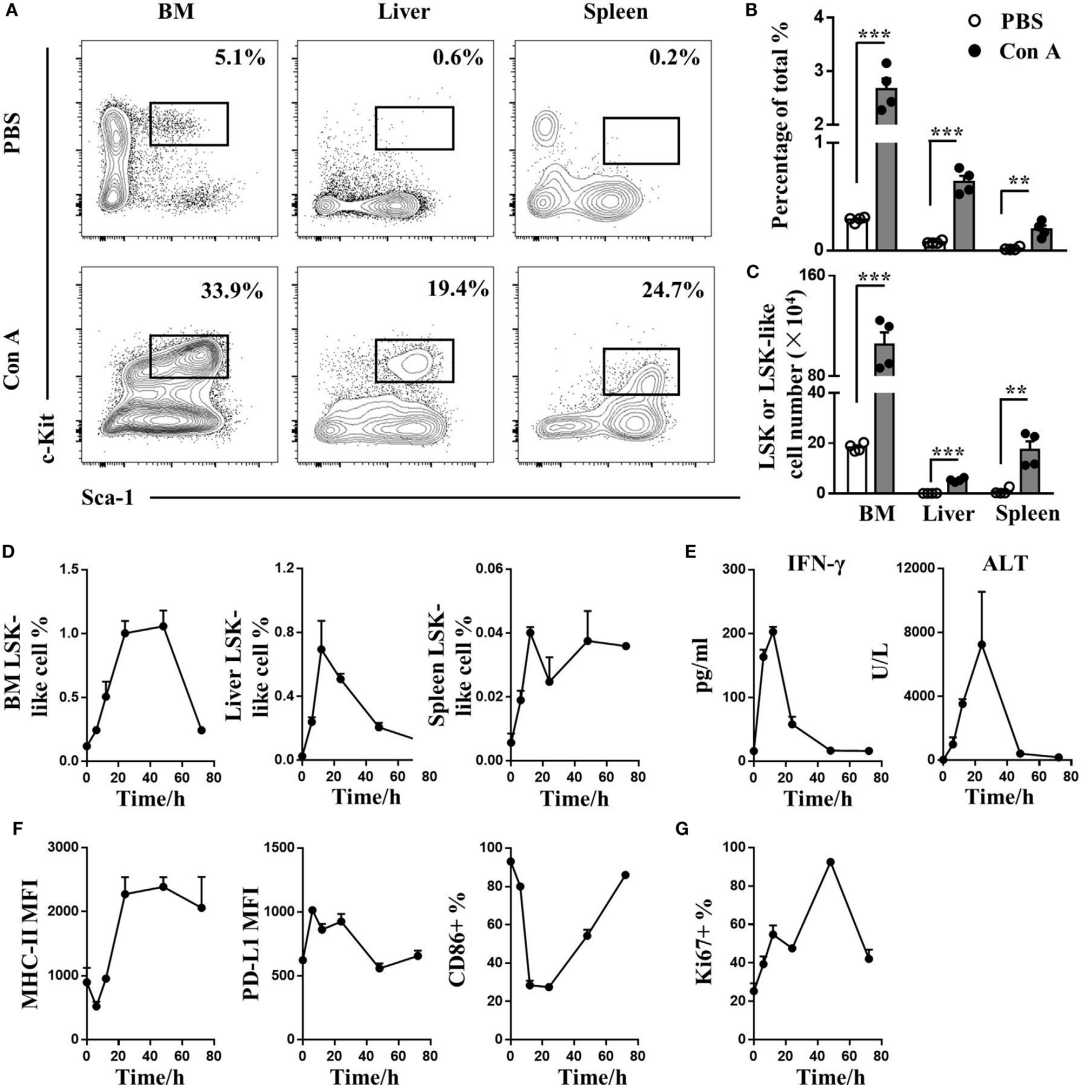
LSK-like cell accumulation during Con A-induced liver AIH. **(A)** Representative flow cytometry result of LSK cells in BM, liver, and spleen of 10-week-old wild type male mice 24 h after injection i.v. with PBS (*n* = 4) and 10 mg/kg Con A (*n* = 4). Numbers represent percentage of lineage^−^ cells. **(B,C)** Statistical analysis of **(B)** percentage and **(C)** number of LSK cells in BM, liver, and spleen of Con A- or PBS-treated mice. **(D,E)** LSK percentage of **(D)** BM, liver, spleen leukocytes, **(E)** serum IFN-γ, and alanine aminotransferase (ALT) level at 0, 6, 12, 24, 48, and 72 h after Con A treatment. **(F)** MHC-II molecule, PD-L1, and CD86 expression of BM LSK-like cells at different time points after Con A injection. **(G)** Percentage of Ki67^+^ BM LSK-like cells at different time point after Con A treatment. *N* = 3 per time point. *p < 0.05, **p < 0.01, ***p < 0.001. Data are shown in mean ± SEM.

In this murine model of Con A-induced autoimmune hepatitis, the frequency (Figures 1A,B) and absolute number (Figure 1C) of LSK-like cells increased in BM, liver, and spleen 24 h after Con A treatment. Specially in BM, percentage of other lineage cells including T, B, NK, NKT, and myeloid cells didn’t change a lot, while their cell number reduced due to a decreased BM total mononuclear cell count (Figure S1A,B in Supplementary Material). Interestingly, LSK cells accumulated in BM, liver, and spleen (Figure 1D) following the increase of serum IFN-γ and ALT (Figure 1E) level, suggesting that LSK-like cell accumulation was induced by inflammatory cytokines. BM LSK-like cells from Con A-treated mice had increased MHC-I/II and PD-L1 but decreased CD86 expression (Figure 1F and data not shown). In addition, they demonstrated a higher proliferation ability after Con A treatment as indicated by Ki67 staining (Figure 1G).

In BM lineage^−^ cells, the percentage of myeloid progenitors (Lin^−^c-Kit^+^Sca-1^−^) decreased a lot in contrast of LSK-like cells (Figure S1C in Supplementary Material). Myeloid progenitors can be divided into three subsets, granulocyte-monocyte progenitor (GMP, Lin^−^c-Kit^+^Sca-1^−^CD34^+^CD16/32^hi^), common myeloid progenitor (CMP, Lin^−^c-Kit^+^Sca-1^−^CD34^+^CD16/32^mid^), and (MKEP, Lin^−^c-Kit^+^Sca-1^−^CD34^−^CD16/32^−^). We found that BM LSK-like cells in Con A-treated mice demonstrated a similar subset distribution as myeloid progenitors in WT mice (Figure S1D in Supplementary Material). Giemsa staining also demonstrated the morphological feature of Con A LSK-like cells resembled WT myeloid progenitors rather than WT LSK cells, such as larger size and lower nucleocytoplasmic tation (Figure S1E in Supplementary Material). Myeloid progenitors have the potential to upregulate Sca-1 expression and develop a LSK-like phenotype under Th1 cytokine stimulation (17). Hence, we sorted myeloid progenitors and cocultured with splenic T cells in the presence of Con A. WT myeloid progenitors could upregulate Sca-1 expression and became LSK-like cells following stimulation of Con A-activated T cells. However, neither IFN-γ^−/−^ T cells co-cultured with WT myeloid progenitors nor WT T cells co-cultured with IFN-γR^−/−^ myeloid progenitors resulted in the induction of LSK-like cells (Figure S1F in Supplementary Material). In addition, IFN-γR^−/−^ myeloid progenitors failed to upregulate Sca-1 expression after IFN-γ stimulation compared to WT myeloid progenitors (Figure S1G in Supplementary Material). Moreover, LSK-like cell accumulation was suppressed in IFN-γ^−/−^, IFN-γR^−/−^ mice, and Rag1^−/−^ mice (Figure S1H in Supplementary Material). These data indicate that LSK-like cells accumulating in Con A-treated mice are myeloid progenitors, which upregulate Sca-1 expression following stimulation of T cell-derived IFN-γ.

### WT Myeloid Progenitors and LSK-Like Cells from Con A-Treated Mice Exhibit Strong T Cell Suppressive Ability *In Vitro*

**Figure 2:**
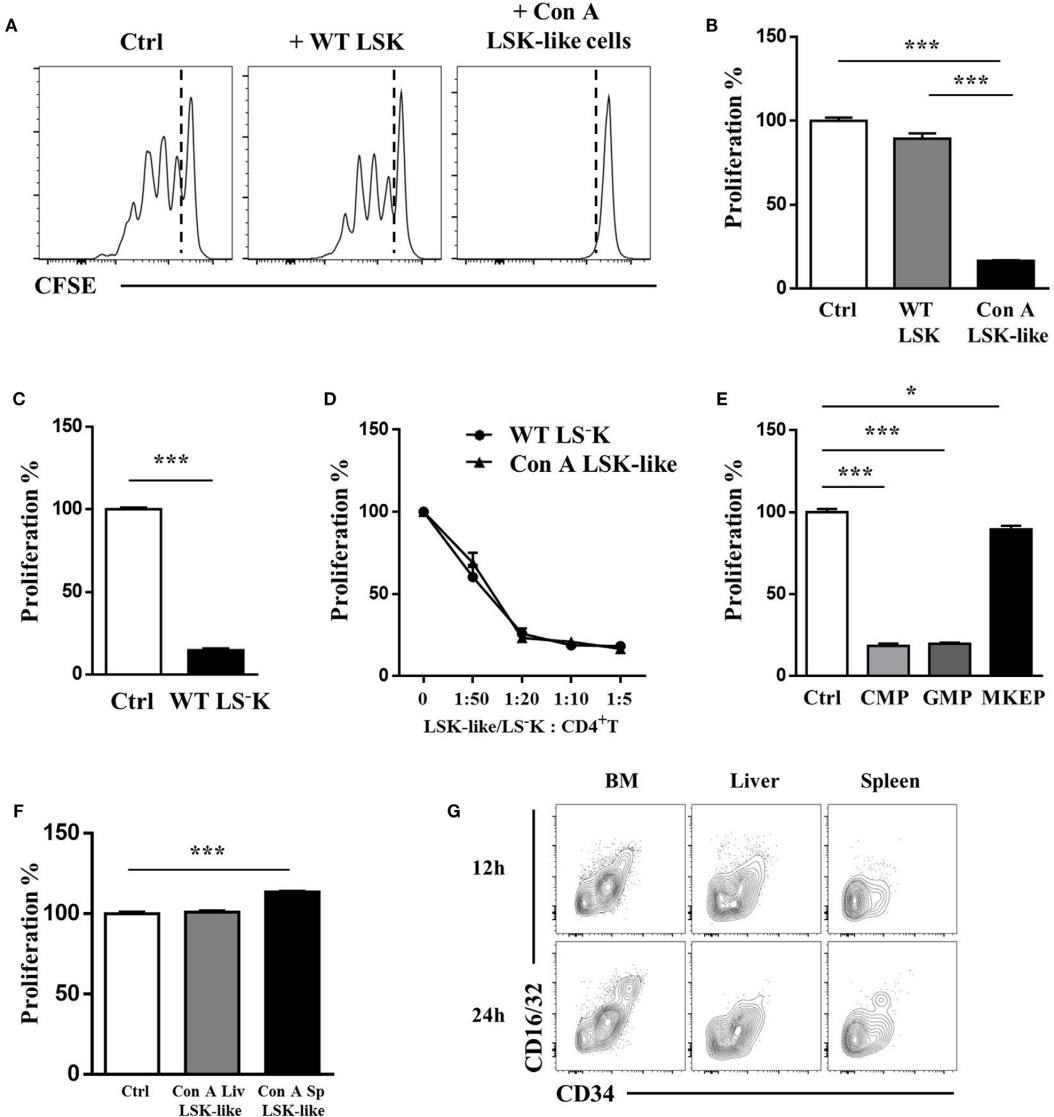
WT LS^−^K cells and LSK-like cells from Con A-treated mice inhibit T cell proliferation *in vitro*. **(A,B)** Proliferation of carboxyfluorescein succinimidyl ester-labeled OT-II T cells (5 × 10^4^/well) cocultured with WT LSK or Con A BM LSK-like cells (1 × 10^4^/well), respectively, for 72 h, in the presence of soluble OVA peptide 323–339 (1 µg/ml) and WT B cells (CD19^+^ cells from spleen of WT mice, 1 × 10^4^/well). **(C)** Proliferation of OVA peptide and B cell-activated OT-II T cells cocultured with WT LS^−^K cells. **(D)** Proliferation of OVA peptide and B cell-activated OT-II T cells cocultured with WT LS^−^K or Con A BM LSK-like cells at indicated ratios. **(E)** The suppression ability of common myeloid progenitors (CMPs), granulocyte-monocyte progenitor (GMP), and megakaryocyte–erythrocyte progenitor subsets, respectively, sorted from WT BM LS^−^K cells. **(F)** The suppression ability of liver and spleen LSK-like cells from mice treated with con A for 24 h. **(G)** Subset of liver, spleen, and BM LSK-like cells from Con A-treated mice at indicated time points. *N* = 3 for coculture experiments and data represent one of at least two independent experiments. *p < 0.05, **p < 0.01, ***p < 0.001. Data are shown in mean ± SEM.

As we observed altered expression of MHC class II, CD86, and PD-L1 on LSK-like cells after Con A treatment or IFN-γ stimulation, we wondered whether these LSK-like cells could regulate T cell responses directly. Hence, we cocultured LSK-like cells from Con A-treated mice with OT-II T cells in the presence of B cells and OVA_323–339_ peptide *in vitro*. Strikingly, BM LSK-like cells from Con A-treated mice almost completely suppressed the proliferation of OT-II T cells, while BM LSK cells from WT mice could not (Figures 2A,B). WT myeloid progenitors exhibited a similar suppressive potential (Figures 2C,D). Interestingly, these LSK-like cells or WT myeloid progenitors were powerful suppressors as there was still a significant inhibition even at the ratio of E:T = 1:50 (Figure 2D). Furthermore, among three subsets of WT myeloid progenitors, GMP and CMP but not MKEP cells exhibited remarkable inhibitory function (Figure 2E). However, the accumulated LSK-like cells in liver or spleen of Con A-treated mice showed no inhibitory function (Figure 2F). This is because these LSK-like cells were mainly MKEP cells, which did not have *in vitro* suppressive activity (Figure 2G). These results demonstrate that WT myeloid progenitors possessed strong *in vitro* T cell suppressive ability.

### Myeloid Progenitors Acquire T Cell Suppressive Function through IFN-γ–STAT1–iNOS Pathway

**Figure 3:**
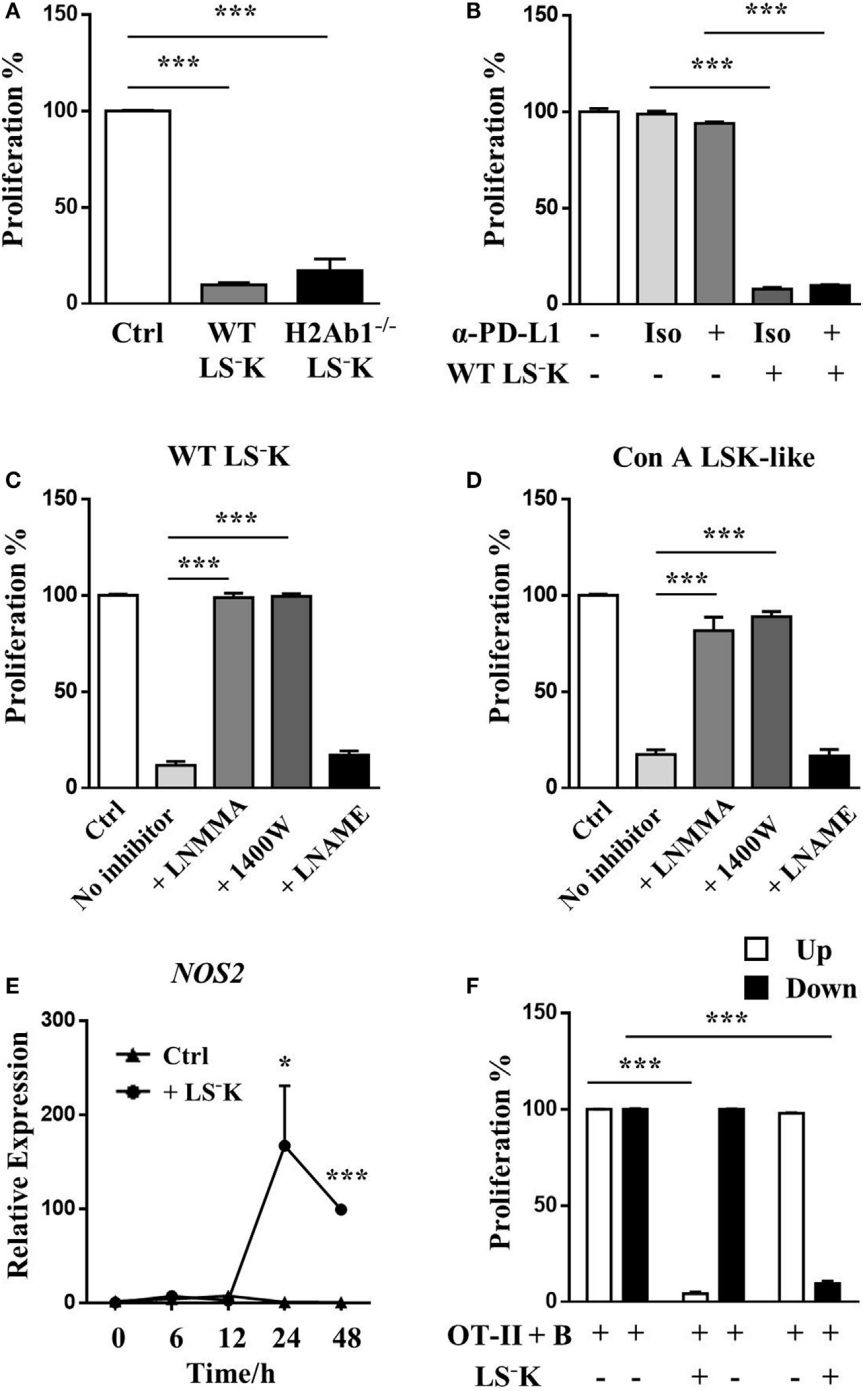
LS^−^K cells suppress T cell proliferation through iNOS activation. **(A)** Proliferation of OVA peptide and B cell-activated OT-II T cells cocultured with WT or H2Ab1^−/−^ BM LS^−^K cells. **(B)** Suppression ability of WT LS^−^K cells in the presence of anti-PD-L1 antibody (5 µg/ml) and isotype control. **(C,D)** Suppression ability of **(C)** WT LS^−^K cells and **(D)** Con A BM LSK-like cells in the presence of L-NMMA [total nitric oxide synthase (NOS) inhibitor], 1,400 W (iNOS inhibitor), or LNAME (eNOS inhibitor). Suppression experiments set as in this figure. **(E)** Expression of iNOS mRNA of WT LS^−^K cocultured with OVA stimulated OT-II T cells after 0, 6, 12, 24, and 48 h. **(F)** Proliferation of OVA peptide and B cell (5 × 10^4^)-activated OT-II T cells (2.5 × 10^5^) cocultured with or without WT LS^−^K cells (5 × 10^4^) in the top or bottom chamber of transwell system. *p < 0.05, **p < 0.01, ***p < 0.001. *N* = 3 for coculture experiments and data represent one of at least two independent experiments. Data are shown in mean ± SEM.

As LSK-like cells induced from myeloid progenitors upregulated MHC-II and downregulated CD86 expression, we first wanted to know whether their suppressive ability was acquired through inducing T cell anergy. Myeloid progenitors from H2Ab1^−/−^ mice, which do not express MHC-II molecules had intact suppressive ability (Figure 3A), indicating that they did not induce T cell anergy during coculture. On the other hand, although LSK-like cells from Con A-treated mice had increased PD-L1 expression, PD-L1 blockade did not influence their suppressive function (Figure 3B). Furtherly, we found the inhibitory function of WT myeloid progenitors and Con A LSK-like cells was iNOS dependent and eNOS independent as they lost their suppressive ability when iNOS was inhibited (Figures 3C,D). Indeed, after coculture with T cells, myeloid progenitors significantly upregulated iNOS expression (Figure 3E). Interestingly, although the suppressive ability of WT myeloid progenitors depends on NO, they failed to transmit through transwell system (Figure 3F).

**Figure 4:**
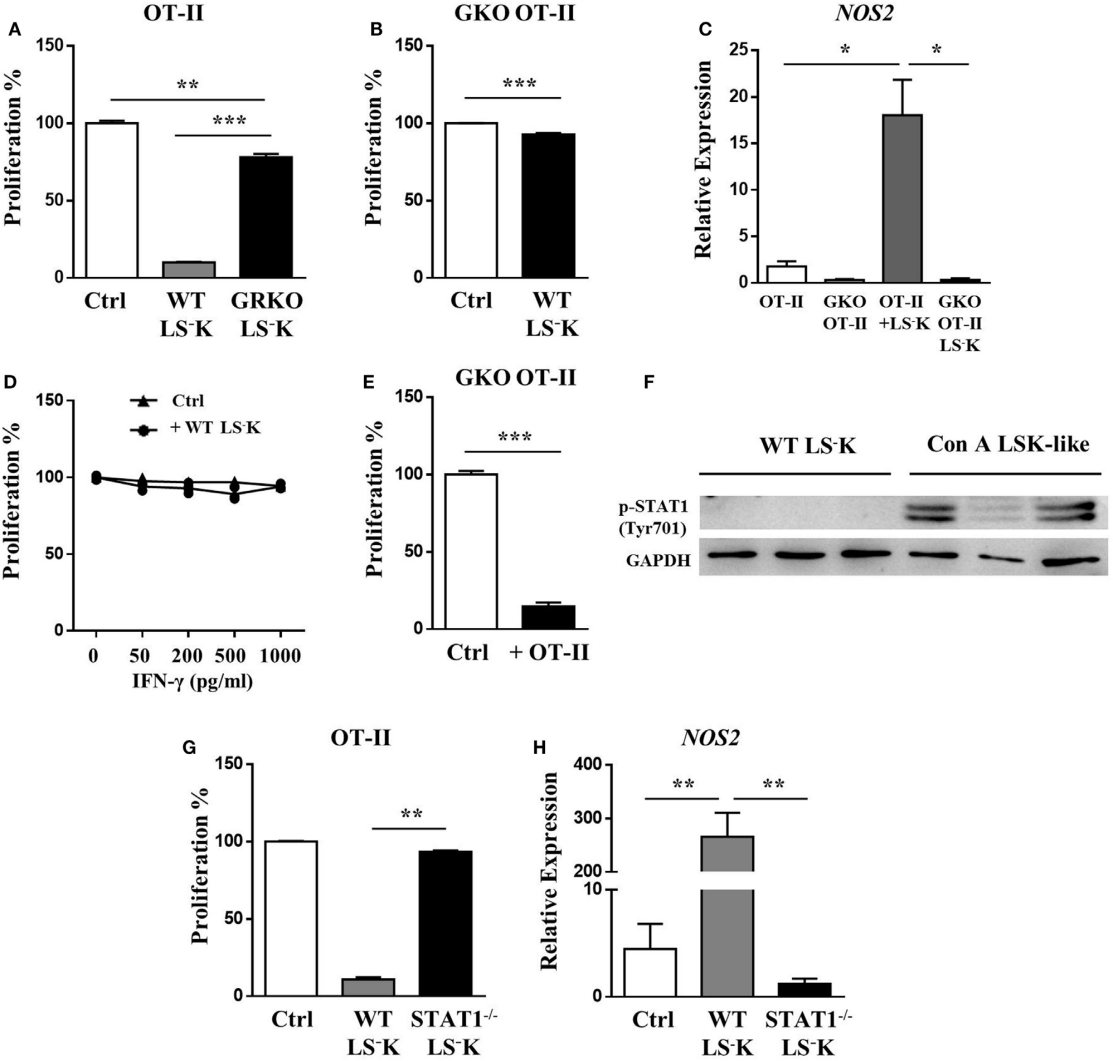
IFN-γ boosts LS^−^K cells suppressive potential *via* STAT-1–iNOS pathway. **(A)** Proliferation of OVA peptide and B cell-activated OT-II T cells cocultured with WT or GRKO LS^−^K cells. **(B)** Suppression ability of WT LS^−^K cells on the proliferation of OVA peptide and B cell-activated GKO OT-II T cells. Suppression experiments set as Figure 3. **(C)** Expression of iNOS mRNA of WT LS^−^K cocultured with OVA-stimulated OT-II T cells or GKO OT-II T cells after 24 h. **(D)** Suppression ability of WT LS^−^K cells on the proliferation of OVA and B cell-activated GKO OT-II T cells under extra IFN-γ administration at indicated concentration. **(E)** Suppression ability of WT LS^−^K cells on the proliferation of OVA peptide and B cell-activated GKO OT-II T cells in the presence of OT-II T cells (1 × 10^4^) or GKO OT-II T cells (1 × 10^4^) as control. Suppression experiments set as Figure 3. **(F)** STAT1 phosphorylation of WT BM LS^−^K cells and BM LSK-like cells from Con A-treated mice. **(G)** Proliferation of OVA peptide and B cell-activated OT-II T cells cocultured with WT or STAT1^−/−^ LS^−^K cells. **(H)** Expression of iNOS mRNA of WT LS^−^K or STAT1^−/−^ LS^−^K cells cocultured with OVA-stimulated OT-II CD4^+^T cells after 24 h. *N* = 3 for coculture experiments and data represent one of at least two independent experiments. *p < 0.05, **p < 0.01, ***p < 0.001. Data are shown in mean ± SEM.

Based on the findings that the accumulation of LSK-like cells after Con A treatment depended on IFN-γ and *NOS2* is one of the IFN-γ stimulated genes, we investigated whether IFN-γ produced by activated T cells was indispensable for the suppressive function of WT myeloid progenitors. Interestingly, IFN-γR^−/−^ myeloid progenitors failed to suppress OT-II T cell proliferation (Figure 4A). Meanwhile, myeloid progenitors could not inhibit the proliferation of IFN-γ^−/−^ OT-II T cells, which could not produce IFN-γ upon OVA peptide stimulation (Figure 4B). WT myeloid progenitors cocultured with IFN-γ^−/−^ OT-II T cells failed to upregulate iNOS expression (Figure 4C). These results indicate that the suppressive ability of myeloid progenitors depends on activated T cell-derived IFN-γ. However, the suppressive function of WT myeloid progenitors could not be restored by addition of IFN-γ (Figure 4D), but by addition of OT-II T cells (Figure 4E), which provided IFN-γ in a paracrine fashion. These results furtherly suggested a cell–cell contact dependent mechanism for the acquirement of suppressive ability. BM LSK-like cells from Con A-treated mice had a higher level of STAT1 phosphorylation than WT myeloid progenitors (Figure 4F). Meanwhile, STAT1^−/−^ myeloid progenitors could not upregulate expression of Sca-1 under IFN-γ stimulation (Figure S1I in Supplementary Material), and they also lost inhibitory function because of defective iNOS induction (Figures 4G,H). These results indicate an IFN-γ–STAT1–iNOS pathway in myeloid progenitor suppressive function responding to T cell activation.

### T Cell Suppressive Ability of Myeloid Progenitors is Independent of Differentiation

**Figure 5:**
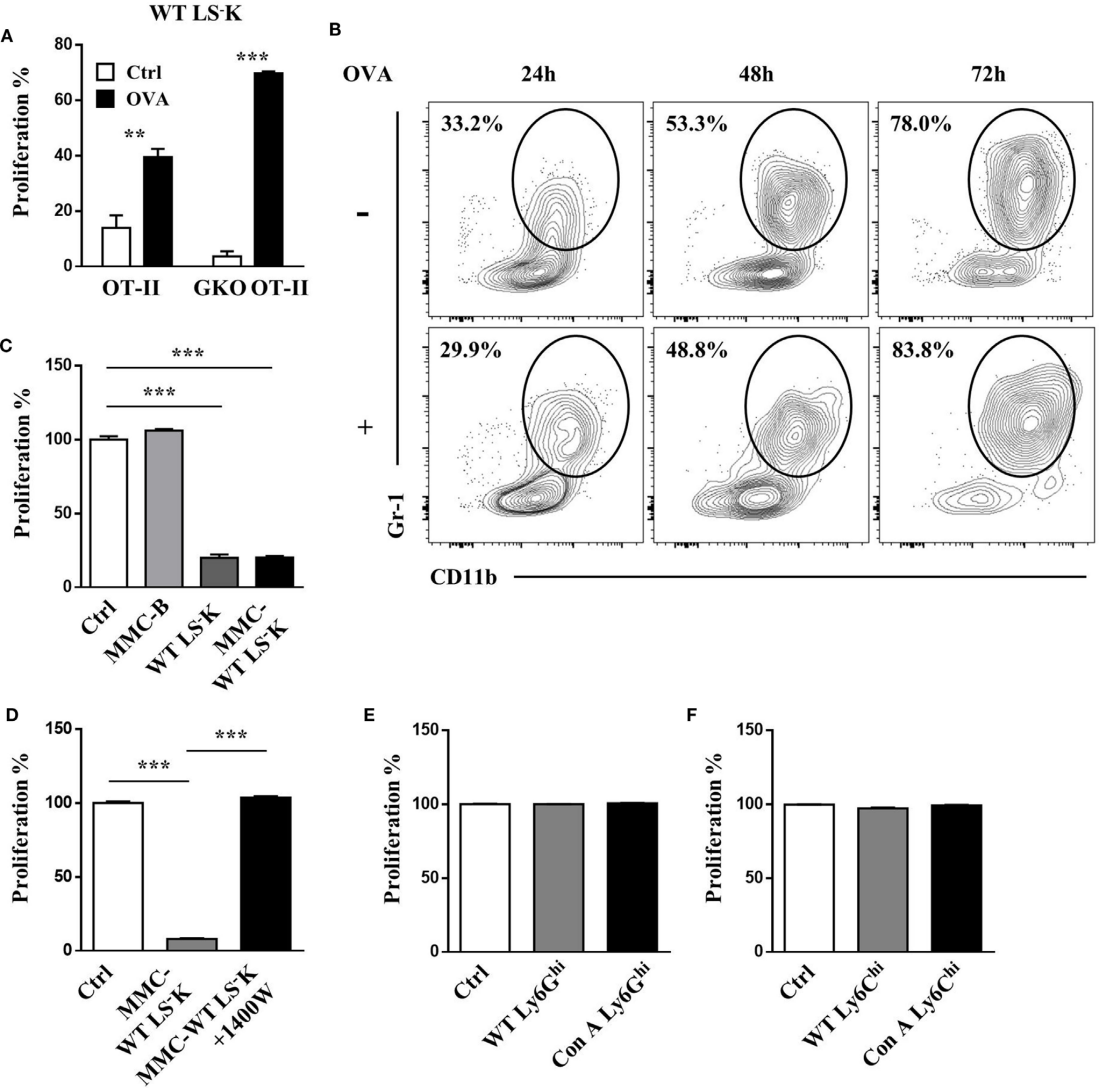
T cell suppressive ability of LS^−^K cells is independent of differentiation. **(A)** Proliferation of WT LS^−^K cells (5 × 10^4^) cocultured with OT-II T cells or GKO OT-II T cells (5 × 10^4^) under OVA (1 µg/ml) stimulation. **(B)** Differentiation of WT LS^−^K cells (5 × 10^4^) cocultured with OT-II T cells (5 × 10^4^) under OVA (1 µg/ml) stimulation or not after 24, 48, and 72 h. **(C)** Proliferation of OVA and B cell-activated OT-II T cells cocultured with mitomycin C-treated WT LS^−^K cells. **(D)** Analyses of the suppression ability of mitomycin C-treated WT LS^−^K cells on OVA and B cell-activated OT-II T cells in the presence of 1,400 W. **(E,F)** Proliferation of OVA and B cell-activated OT-II T cells cocultured with **(E)** CD11b^+^ly6G^hi^ cells and **(F)** CD11b^+^ly6C^hi^ cells from spleen of WT or Con A-treated mice. Suppression experiments set as Figure 3. *p < 0.05, **p < 0.01, ***p < 0.001. *N* = 3 for coculture experiments and data represent one of at least two independent experiments. Data are shown in mean ± SEM.

During coculture with T cells, WT myeloid progenitors could be activated and undergo proliferation (Figure 5A) with upregulation of CD11b and Gr-1 expression (Figure 5B), hence presenting a myeloid-like phenotype. However, the expression of CD11b and Gr-1 was not associated with activated T cell stimulation because myeloid progenitors acquired similar phenotype when cocultured with non-stimulated T cells (Figure 5B). Mitomycin C treatment, which could block cell proliferation and differentiation, did not impair suppressive function of myeloid progenitors (Figure 5C), which was still dependent of iNOS (Figure 5D). Besides, we sorted CD11b^+^Ly6G^hi^ cells and CD11b^+^Ly6C^hi^ cells from the BM and spleen of WT and Con A-treated mice and found that they could not inhibit the proliferation of T cells as myeloid progenitors (Figures 5E,F and data not shown). These results indicate that the inhibitory ability of myeloid progenitors was independent of differentiation.

## Discussion

Our present work demonstrates the suppressive potential of myeloid progenitors and reveals the mechanism that leads to this ability. Different from a previous report that early myeloid progenitors can be immunosuppressive cells in a NO-dependent way (18), we used a more specific coculture system, which may be implicated in suppression of antigen-specific T cell activation. We also demonstrated a mechanism of inflammation-induced suppressive function of myeloid progenitors.

Inflammatory conditions such as found in a tumor microenvironment can affect HSPC development and function (4, 6). In cancer patients, circulating HSPCs are myeloid-biased, and tumor cells can induce early myeloid differentiation into immunosuppressive neutrophils, which resemble granulocytic myeloid-derived suppressor cells (MDSCs) (19, 20). MDSCs are described as a heterogeneous population of myeloid progenitor cells and immature myeloid cells, which have remarkable ability to suppress T cell response especially in tumor (21, 22). It is reported that tumor-derived GM-CSF are sufficient to induce MDSCs from myeloid progenitors, but the suppressive function of these tumor-induced MDSCs was independent of IFN-γ (23, 24). Myeloid dendritic cell precursors appear transiently during BM cell culture with GM-CSF and can suppress T cell response *in vitro* in a cell contact and NO-dependent way (25). Without the signals provided by BM niche, we observed spontaneous differentiation of myeloid progenitors *in vitro* with the phenotype of CD11b^+^Gr-1^+^, resembling granulocytic MDSCs. However, their differentiation was not affected by coculture with T cells, or by IFN-γ stimulation. Importantly, mitomycin C-treated myeloid progenitors, which lost proliferation and differentiation ability maintained *in vitro* suppressive function. Our data demonstrate that myeloid progenitors acquire T cell suppressive function under the effect of inflammatory cytokine IFN-γ without dependency of differentiation into mature or immature myeloid cells and were different from MDSCs.

Antigen-specific CD4^+^ T cells could convert immunosuppressive MDSCs into powerful non-specific suppressor cells, which depend on cell–cell contact and crosslinking of MHC class II molecule on MDSCs (26). We also found increased MHC class II molecule expression on LSK-like cells after ConA treatment, as well as after coculture with T cells. Besides, they showed decreased costimulatory molecule CD86 expression. Based on the *in vitro* suppressive function of these LSK-like cells and myeloid progenitors, our initial hypothesis was that they could induce T cell anergy by providing “signal 1” without “signal 2” during T cell activation. However, myeloid progenitors with MHC class II knockout maintained *in vitro* suppressive function. Also, addition of extra anti-CD28, which provide “signal 2” did not impair their suppressive ability *in vitro* (data not shown). IFN-γ can induce the expression of MHC class II, PD-L1, and CD86 (27–29). However, block of PD-1–PD-L1 interaction did not impair the suppressive function of myeloid progenitors. We found that myeloid progenitors express very low level of CD86. After IFN-γ stimulation, they shifted to LSK-like cells, but did not upregulate CD86 expression, resulting in decreased CD86 expression. These results indicated that increased MHC class II and PD-L1, and decreased CD86 expression of LSK-like cells during AIH were not associated with their interaction with T cells.

IFN-γ can activate HSPCs and induce expansion of LSK cells, as well as a myeloid-biased differentiation of HSPCs (17, 30). However, chronic IFN-γ stimulation inhibits the generation of myeloid progenitors and prevents myeloid lineage differentiation (31). In our study, we found that T cell-derived IFN-γ functioned through IFN-γRI on myeloid progenitors, which induced the phosphorylation and translocation of STAT1 to the nucleus. Further, p-STAT1 induced the expression of iNOS and the production of NO, which suppressed T cell activation. On the other hand, myeloid progenitors cocultured with GKO OT-II T cells have a higher proliferation ratio than with OT-II T cells, suggesting that IFN-γ suppressed their proliferation *in vitro*. However, although extra IFN-γ administration did not impair their suppressive ability on OT-II T cells, it could barely restore their suppressive ability on GKO OT-II cells. Thus, we suggested that the suppressive ability of myeloid progenitors may depend on a close contact with T cells and the pulse stimulation of IFN-γ. But whether they can maintain suppressive ability during chronic inflammation needs further investigation.

Although IFN-γ stimulated myeloid progenitors suppressed T cells in a NO-dependent way, our result showed that their suppressive ability failed to transmit through a transwell system. Low dose of NO is reported to promote type I T cell differentiation, while high dose of NO inhibited the proliferation of T cells (32). On the other hand, NO diffuses quickly from its source (33, 34), but the concentration of the active form drops sharply within about 100 mm (35). Therefore, NO can act only in close proximity to the cells producing it. In our antigen-specific coculture system, the number of myeloid progenitors may not be enough to generate sufficient NO to transmit through a transwell system. Besides, NO may be consumed by T cells cocultured with myeloid progenitors.

Bone marrow is thought to be an immune privileged site, in which stromal cells and Treg cells compose a tolerated niche to protect HSPCs from environmental insults (36, 37). Interestingly, BM is also a major reservoir and site of recruitment for memory T cells and a preferential homing site for autoreactive T cells in type I diabetes (38, 39). In our work herein, we found myeloid progenitors could suppress antigen-specific T cell activation *in vitro*. T cells in the BM are reported to locate near stromal cells, as well as HSPCs (40). Thus, we raise the thesis of whether myeloid progenitors can interact with BM-resident memory T cells and act as vanguard to maintain immune homeostasis in BM. During antigen challenge, memory T cells could be rapidly activated and produce IFN-γ. These HSPCs activated by IFN-γ could suppress further activation of these memory T cells and maintain homeostasis in the BM. This process reflects an interaction between an autoimmune response and hematopoietic system regulation, which has the potential to allow hematopoietic system to influence immune tolerance.

However, liver inflammation was not suppressed by accumulated LSK-like cells in Con A-induced liver injury. On one hand, these LSK-like cells in the liver were mainly MKEP cells, which did not have suppressive function upon T cell-derived IFN-γ stimulation. On the other hand, IFN-γ is thought to be pro-inflammatory in the autoimmune phase of Con A-induced liver injury (41), while their level decreased quickly after 24 h. Moreover, the suppressive activity requires close contact of myeloid progenitors. Thus, other models of autoimmunity with chronic IFN-γ production and transfer experiments may be needed to fully understand the *in vivo* suppressive function of myeloid progenitors.

In conclusion, we dissect the direct interaction between HSPCs and T cells and our data demonstrate a mechanism of inflammation induced suppressive function of myeloid progenitors, which may participate directly in suppressing T cell-mediated immunopathology.

## Ethics Statement

This study was carried out in accordance with the recommendations of Guide for the Care and Use of Laboratory Animals, USTC Animal Care and Use Committee. The protocol was approved by the USTC Animal Care and Use Committee.

## Author Contributions

S-HY, LL, YY, H-DM, and Z-XL: design of the work. S-HY and LL: experimental work, the acquisition, analysis, and interpretation of data for the work; Y-QX, YY, C-YG, and L-HL: experimental work. H-DM, MG, and Z-XL: final approval of the version to be published.

## Conflict of Interest Statement

The authors declare that the research was conducted in the absence of any commercial or financial relationships that could be construed as a potential conflict of interest.

## Abbreviations

AIH, autoimmune hepatitis; BM, bone marrow; CMP, common myeloid progenitor; Con A, Concanavalin A; GKO, IFN-γ knock out; GM-CSF, granulocyte-macrophage colony-stimulating factor; GMP, granulocyte-monocyte progenitor; GRKO, IFN-γ receptor knock out; HSPCs, hematopoietic stem and progenitor cells; LSK, lineage^−^Sca-1^+^c-Kit^+^; MDSCs, myeloid-derived suppressor cells; MKEP, megakaryocyte–erythrocyte progenitor; NO, nitric oxide; NOS, nitric oxide synthase; WT, wild type.
