# Supplementary material for: IFN-γ–STAT1–iNOS Induces Myeloid Progenitors to Acquire Immunosuppressive Activity
Source: Front Immunol. 2017 Sep 22;8:1192. doi: 10.3389/fimmu.2017.01192 (PMC5614959; doi:10.3389/fimmu.2017.01192)
Supplement: Supplementary file 1 [file data_sheet_1.docx]

Supplementary Material

IFN-γ-STAT1-iNOS Induces Myeloid Progenitors to Acquire T Cell Suppressive Activity

Shu-Han Yang,^1,2, #^ Liang Li, ^1,2, #^ Yu-Qing Xie,^1^ Yuan Yao,^1,2^ Cai-Yue Gao,^1,2^ Liang-Huan Liao,^1,2^ Hong-Di Ma,^1^ M. Eric Gershwin^3^ and Zhe-Xiong Lian^1,2,4,^ *

^1^Liver Immunology Laboratory, Institute of Immunology and School of Life Sciences, University of Science and Technology of China, Hefei 230027, China;

^2^Chronic Disease Laboratory, Institutes for Life Sciences and School of Medicine, South China University of Technology, Guangzhou 510006, China;

^3^Division of Rheumatology, Allergy and Clinical Immunology, University of California at Davis School of Medicine, Davis, CA, USA;

^4^Innovation Center for Cell Signaling Network, Hefei National Laboratory for Physical Sciences at Microscale, Hefei 230027, China.

^#^ Both authors contributed equally to the manuscript.

***Correspondence to**: Zhe-Xiong Lian, M.D., Ph.D., Liver Immunology Laboratory, Institute of Immunology and School of Life Sciences, University of Science and Technology of China, Hefei 230027, China; Phone: +86-551-63600317; e-mail: [zxlian1@ustc.edu.cn](mailto:zxlian1@ustc.edu.cn).

# Supplementary Figures and Tables

## Supplementary Figures


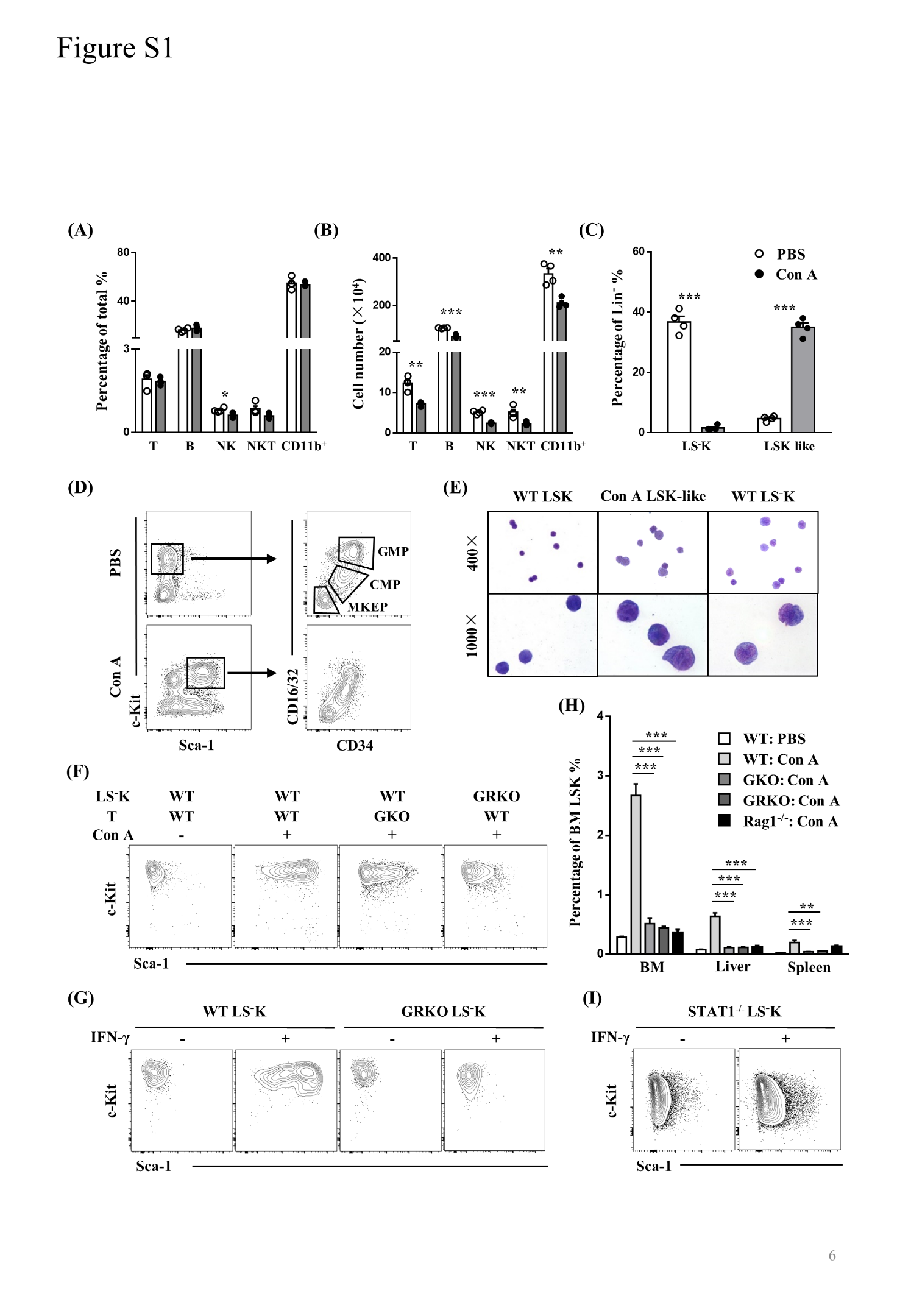


**Supplemental figure 1. T cell-derived IFN-γ induces LSK cells from LS^-^K cells.**

**(A-B)** Statistical analysis of **(A)** percentage and **(B)** Number of T, B, NK, NKT and CD11b^+^ cells in BM of Con A (n=4) or PBS (n=4) treated mice. **(C)** Percentage of LS^-^K and LSK-like cells of lineage- cells in BM of Con A or PBS treated mice. **(D)** Subsets of WT BM LS^-^K cells and Con A BM LSK cells. **(E)** Morphological features of WT LSK, Con A BM LSK and WT LS^-^K cells by Giemsa staining, original magnification ×400 and ×1000. **(F)** Expression of Sca-1 on WT LS^-^K cells (co-cultured with WT or GKO splenic T cells ) and GRKO LS^-^K cells (co-cultured with WT splenic T cells) in the presence or absence of Con A (2μg/ml) for 24h. **(G)** Expression of Sca-1 on WT LS^-^K cells and GRKO LS^-^K cells treated with 20ng/ml IFN-γ for 24h. **(H)** Percentage of LSK cells in BM, liver and spleen of Con A treated WT (n=4), GKO (n=4) and GRKO (n=4), Rag1^-/-^ mice (n=3) and WT PBS control (n=4). **(I)** Expression of Sca-1 on STAT1^-/-^ LS^-^K cells treated with 20ng/ml IFN-γ for 24h. Data represent one of at least two independent experiments. *p < 0.05, **p < 0.01, ***p < 0.001. Data are shown in mean ± SEM.
